# Accuracy of Three Serological Techniques for the Diagnosis of Imported Schistosomiasis in Real Clinical Practice: Not All in the Same Boat

**DOI:** 10.3390/tropicalmed8020073

**Published:** 2023-01-19

**Authors:** María Pilar Luzón-García, María Isabel Cabeza-Barrera, Ana Belén Lozano-Serrano, Manuel Jesús Soriano-Pérez, Nerea Castillo-Fernández, José Vázquez-Villegas, Jaime Borrego-Jiménez, Joaquín Salas-Coronas

**Affiliations:** 1 Tropical Medicine Unit, Hospital Universitario Poniente, Ctra. de Almerimar 31, 04700 El Ejido, Spain; 2 Tropical Medicine Unit, Distrito Poniente de Almería, 04700 El Ejido, Spain; 3 Department of Nursing, Physiotherapy and Medicine, Faculty of Health Sciences, University of Almería, 04120 La Cañada, Spain

**Keywords:** schistosomiasis, *Schistosoma haematobium*, *Schistosoma mansoni*, diagnosis, serology, immunochromatography, migrants

## Abstract

Schistosomiasis is a neglected tropical disease despite of being a major public health problem affecting nearly 240 million people in the world. Due to the migratory flow from endemic countries to Western countries, an increasing number of cases is being diagnosed in non-endemic areas, generally in migrants or people visiting these areas. Serology is the recommended method for screening and diagnosis of schistosomiasis in migrants from endemic regions. However, serological techniques have a highly variable sensitivity. The aim of this study was to evaluate retrospectively the sensitivity of three different serological tests used in real clinical practice for the screening and diagnosis of imported schistosomiasis in sub-Saharan migrant patients, using the detection of schistosome eggs in urine, faeces or tissues as the gold standard. We evaluated three different serological techniques in 405 sub-Saharan patients with confirmed schistosomiasis treated between 2004 and 2022: an enzyme-linked immunosorbent assay (ELISA), an indirect haemagglutination assay (IHA) and an immunochromatographic test (ICT). The overall sensitivity values obtained with the different techniques were: 44.4% for IHA, 71.2% for ELISA and 94.7% for ICT, respectively. According to species, ICT showed the highest sensitivity (*S. haematobium*: 94%, *S. mansoni*: 93.3%; and *S. intercalatum*/*guineensis*: 100%). In conclusion, our study shows that Schistosoma ICT has the best performance in real clinical practice, when compared to ELISA and IHA, in both *S. mansoni* and *S. haematobium* infections.

## 1. Introduction

Schistosomiasis is a neglected tropical disease despite of being a major public health problem affecting nearly 240 million people in at least 78 countries, with more than 700 million living in areas at risk of infection [1,2,3]. Ninety-three per cent of cases occur in sub-Saharan Africa. It is caused by blood flukes of the genus *Schistosoma*. Seven species can infect humans, although most cases are caused by *S. haematobium* and *S. mansoni*, responsible for urogenital and intestinal schistosomiasis, respectively [4]. Human infections with the remaining species are much less frequent and are restricted to their intermediate host distribution [5,6]. The disease can lead to serious complications causing the death of about 300,000 people each year.

The significant migratory flow experienced in recent decades from endemic countries to Western countries has led to an increase in the diagnosis of imported diseases. These diagnoses may be delayed by language barriers, bureaucracy, access to health care, lack of knowledge of the disease amid health care personnel, poor symptomatology or diagnostic difficulties, among other causes [7]. Regarding schistosomiasis, an increasing number of publications present series of cases diagnosed in non-endemic areas [8,9,10,11,12,13], generally in migrants or people visiting these areas. On the other hand, outbreaks of autochthonous transmission of *S. haematobium* have recently been described in Corsica (France) and Almería (Spain) [14,15].

In 2019, the European Centre for Disease Prevention and Control (ECDC) recommended serological screening for schistosomiasis in migrants from endemic regions staying in Europe for less than 5 years [3].

Although direct visualisation of *Schistosoma* spp. eggs by microscopic examination of urine and faeces is considered the gold standard for diagnosis, it has limitations, such as low sensitivity, especially in non-endemic areas or in cases of acute infection, and the need for experienced personnel [6]. Antigen detection tests, such as circulating anodic antigen (CAA) and circulating cathodic antigen (CCA), and molecular tests (polymerase chain reaction [PCR] or loop-mediated isothermal amplification of nucleic acids [LAMP]) are more sensitive and potentially useful for the diagnosis of schistosomiasis at all stages and for treatment evaluation [5], but their use is often limited to reference centres [16,17,18,19].

Serological tests are currently considered the most effective method for the detection of schistosomiasis in low endemicity and low prevalence settings as they are more sensitive compared to traditional parasitological methods, although they cannot differentiate between active or past disease, and in cases of acute infection, antibodies develop within 6–8 weeks after primary infection [17,20]. Numerous serological techniques, such as indirect haemagglutination (IHA), indirect immunofluorescence assay (IFAT), enzyme-linked immunosorbent assay (ELISA) or immunochromatography-based rapid diagnostic tests (ICT), are commercially available. Most use antigens specific to *S. mansoni* at different stages of the life cycle (adult worm, soluble egg, cercarial antigens, etc.) although these proteins are sufficiently similar to diagnose infections by other *Schistosoma* species with high sensitivity [17,18]. One of the drawbacks of serological testing is that the different techniques have a highly variable sensitivity [21,22,23] and that is why the correct choice of a particular technique is of great importance, especially for screening programmes.

The aim of this study is to evaluate the sensitivity of three different serological techniques used in real clinical practice for the screening and diagnosis of imported schistosomiasis in sub-Saharan migrant patients, using the detection of schistosome eggs in urine, faeces or tissues as the gold standard.

## 2. Materials and Methods

A retrospective observational study of sub-Saharan migrant patients with parasitologically confirmed schistosomiasis treated at the Tropical Medicine Unit (TMU) of the Poniente University Hospital (El Ejido, Almería, Spain) from September 2005 to July 2022 was conducted. The Poniente area is an administrative area located in Southeast Spain holding a population close to 300,000 inhabitants with migrants accounting for 21% of the population, many of them coming from sub-Saharan countries to work in horticultural greenhouses.

In order to attend to such residents, there is a screening protocol in Primary Care consisting in a series of laboratory tests aimed to detect imported and cosmopolitan diseases. The tests are usually offered the first-time migrants contact the public health care system, no matter the reason they consult for. For sub-Saharan migrants, the screening protocol includes blood count, liver and renal function tests, syphilis, HIV, HBV and HCV serologies, tuberculin skin test and search for stool parasites and urine parasites.

Whenever an imported disease is either suspected or diagnosed, migrant patients are referred to the hospital Tropical Medicine Clinic. The hospital’s protocol is ampler and comprises medical history, epidemiological data, complete physical examination and several additional tests: *Strongyloides* and *Schistosoma* serologies, and Knott and/or saponin tests for microfilariae. Chest and abdominal X-rays are routinely performed too. If any other specific disease is suspected (e.g., onchocerciasis, malaria, etc.), further proper diagnostic procedures are performed.

Parasitological diagnosis of schistosomiasis was made by microscopic visualisation of *S. mansoni* and *S. intercalatum*/*guineensis* eggs in stool samples (Ritchie’s technique in three samples collected on alternate days); of *S. haematobium* eggs in urine (10 mL. of a single sample obtained ideally 10–14 h after light physical exercise), or detection of *Schistosoma* spp. eggs in biopsy samples from different tissues, mainly bladder and rectum.

According to organ involvement, we classified schistosomiasis as hepatointestinal (HI) if *S. mansoni* or *S. intercalatum*/*guineensis* eggs were demonstrated in faeces or *Schistosoma* spp. eggs were seen in rectal or appendicular biopsy. Urogenital schistosomiasis (UG) was considered when *S. haematobium* eggs were visualized in urine or *Schistosoma* spp. eggs were detected in bladder, cervix or testicular biopsy.

Throughout the study period, three different commercial serological tests were used to detect antibodies to *Schistosoma* spp. in the sera of the study patients: (i) an enzyme-linked immunosorbent assay (ELISA), *Schistosoma mansoni* IgG-ELISA (NovaLisaTM. NovaTec Immundiagnostica, Dietzenbach, Germany); (ii) an indirect haemagglutination test (Bilharziose Fumouze IHA. Famouze Diagnostics, Levallois-Perret, France); (iii) an immunochromatographic rapid diagnostic test (Schistosoma ICT IgG-IgM^®^. LDBIO Diagnostics, Lyon, France) that simultaneously detects IgM and IgG antibodies against *Schistosoma* spp.

For antibody detection, *S. mansoni* IgG-ELISA NovaLisa TM used a soluble worm antigen preparation [22]. Results were interpreted as negative (index < 1), indeterminate (index 1–1.1) and positive (index > 1.1). For indirect haemagglutination, sensitized sheep erythrocytes coated with *S. mansoni* adult worm antigen were used [24] and a titre ≥1:160 was considered positive. For the Schistosoma ICT immunochromatographic test, nitrocellulose strips were coated with an antigen purified from crude lysate of adult *S. mansoni* worms. The test was considered positive if both the control and test strips were positive [25].

The use of one technique or another was determined by their availability in our laboratory. From 2005 to 2013 and from 2015 to 2017, serum samples were evaluated by *Schistosoma mansoni* IgG ELISA (NovaLisaTM). During 2014, the technique used was indirect haemagglutination (Famouze Diagnostics, Levallois-Perret, France). As of January 2018, *Schistosoma* spp. serology was performed in our centre using the Schistosoma ICT IgG-IgM^®^ immunochromatographic test (LDBIO Diagnostics).

We defined the sensitivity of the technique as the proportion of patients with a positive test result among those with a parasitologically proven infection. Schistosomiasis was considered confirmed when *Schistosoma* spp. eggs were detected in faeces, urine or tissues.

A descriptive statistical analysis was performed including all patients diagnosed with confirmed schistosomiasis. Quantitative variables were expressed as mean ± standard deviation or median ± interquartile range. Qualitative variables were expressed as frequencies and percentages. Data were analysed using the statistical software package SPSS v17.

## 3. Results

A total of 405 sub-Saharan migrants with confirmed schistosomiasis were included in the study. Table 1 shows the epidemiological, clinical and analytical characteristics of the patients. The majority (93.6%) were men with a mean age of 27 years (11–52). The mean length of stay in Spain was 35.7 months (1–288). The main countries of origin were Mali (n = 199; 49%), Senegal (n = 92; 22.7%) and Mauritania (n = 33; 8.1%). The most frequent reasons for referral to the TMU were macroscopic hematuria (29.1%), abdominal pain (26.4%) and eosinophilia (16.8%). The rest of the patients were referred because of reasons other than schistosomiasis, mainly because of chronic hepatitis B.

(1) 5 patients had co-infection with *S. mansoni* and 1 co-infection with *S. mansoni* and *S. intercalatum*/*guineensis*. Two patients also had positive rectal biopsies.

(2) Detection of *Schistosoma* eggs in biopsies from urogenital region: 35 in bladder biopsy (one of them also with *S. intercalatum*/*guineensis* in faeces), 2 in testicular biopsies, 1 in cervix biopsy.

(3) 1 patient had co-infection with *S. mansoni*, 1 with *S. haematobium* and *S. mansoni*, and 1 had a bladder biopsy showing *Schistosoma* eggs.

(4) Detection of *Schistosoma* eggs at hepatointestinal level: 24 in rectal biopsy (2 of them in addition with *S. haematobium*) and 2 in appendiceal biopsy.

In 263 patients (64.9%), *S. haematobium* eggs were demonstrated in urine, 79 (19.5%) had *S. mansoni* eggs in faeces and 10 (2.5%) had *S. intercalatum*/*guineensis* eggs in faeces. Thirty-five patients were diagnosed after the detection of *Schistosoma* spp. eggs in bladder biopsies, 24 in rectal biopsies, 2 in testicular biopsies, 2 in appendicular biopsies and 1 in cervical biopsy. 

Co-infection of different *Schistosoma* species was observed in 10 patients: 5 with *S. haematobium-S. mansoni* co-infection; 1 *S. intercalatum/guineensis-S. mansoni*; 1 *S. haematobium-S. mansoni-S. intercalatum/guineensis*; 1 patient had *Schistosoma* spp. eggs in bladder biopsy and *S. intercalatum*/*guineensis* in faeces; and 2 patients had *Schistosoma* spp. eggs in rectal biopsy and *S. haematobium* in urine.

Overall, serology was positive in 76% of cases (308/405) (Table 2). ELISA was used in 302 patients (74.6%), IHA in 9 (2.2%) and ICT in 94 (23.2%). Sensitivity values ranged from 44.4% for IHA to 94.7% for ICT/LDBIO. Serology was negative in 97 patients: 71 had *S. haematobium* infection, 11 had *S. mansoni* infection, one had triple *S. haematobium-S. mansoni-S. intercalatum/guineensis* co-infection and 14 were patients diagnosed by biopsy (10 bladder, 3 rectal and one cervical).

To calculate the sensitivity of serological tests according to urogenital or hepatointestinal involvement, only patients with infection in one of the locations were considered. The results are shown in Table 3. For urogenital schistosomiasis, the sensitivity was 28.6% for IHA, 66.2% for ELISA/NovaLisaTM and 94.2% for ICT/LDBIO. For hepatointestinal schistosomiasis, sensitivities were 100% for IHA, 84.3% for ELISA/NovaLisaTM and 95% for ICT/LDBIO, respectively.

To determine sensitivity according to *Schistosoma* species (Table 4), only patients with monoinfections were considered, also excluding patients with biopsy-diagnosed schistosomiasis. For *S. haematobium* the sensitivity was 28.6% with IHA, 65.7% with ELISA/NovaLisaTM and 94% with ICT/LDBIO. For *S. mansoni* the sensitivity was 100% for IHA, 82.1% for ELISA/NovaLisaTM and 93.3% for ICT/LDBIO. For *S. intercalatum*/*guineensis* the sensitivity was 100% for all techniques.

## 4. Discussion

Based on the results of our study, we can affirm that ICT is the serological method analysed that shows the best sensitivity for the diagnosis of imported schistosomiasis in sub-Saharan migrants, both urogenital and hepatointestinal, in real clinical practice. As it is a rapid test, thus easy to perform and interpret, it could be recommended as a screening test in non-endemic regions.

Studies on the prevalence of schistosomiasis in migrants from endemic areas in Europe are scarce and show figures of seroprevalence much higher than those obtained by direct microscopy. Serre et al. reported a prevalence of schistosomiasis by microscopic examination of 9% in migrants living in shelters in Barcelona [26]. Salas-Coronas et al. found that in newly arrived African migrants in Spain, a direct diagnosis of schistosomiasis was made in 12.3% of the subjects while the seroprevalence was 32.2% [9]. In a similar study in Italy in refugees, 17.4% were diagnosed by microscopy and serology (ELISA) was positive in 27.6% of cases [27]. In a German study in unaccompanied minors, *Schistosoma* spp. eggs were visualized in 24.7% of cases [28]. In view of these data, we can state that despite the low sensitivity of microscopy, the prevalence of schistosomiasis in sub-Saharan migrants recently arrived in Europe is high.

The sensitivity of the different commercially available serological tests varies significantly depending on the technique used and the population under study. In relation to IHA, although the number of patients studied was small, we found a low sensitivity (44.4%) for the detection of *Schistosoma* spp. infection, which coincides with other studies such as that of Leblanc et al. that showed a sensitivity of 48% in a study conducted in children coming from an endemic area in the previous 12 months or in autochthones after a stay of at least 3 months in an endemic area and skin contact with fresh water during the journey [10]. Additionally, Yameny, in a cross-sectional study in Egypt designed to evaluate the efficacy of this technique compared to microscopy, obtained a 42% sensitivity using 50 *S. haematobium* positive samples and 50 negative ones [29]. However, Hinz et al., in a literature review, presenting performance data from a wide range of serological techniques, found sensitivity values of 73–94% for IHA Fumouze Diagnostics in imported schistosomiasis in travellers [21]. Kinkell et al., using frozen sera from 121 patients with various parasitic infections (with 37 cases of schistosomiasis among them) and 20 sera samples from healthy volunteers, obtained a sensitivity of 73% [22]. Van Gool et al., in a study evaluating various serological tests for the diagnosis of imported schistosomiasis in patients who had recently visited an African country endemic for schistosomiasis, reported a sensitivity for IHA of 86% and 94% depending on whether they considered the cut-off titre of 1:160 (suggested by the manufacturer) or 1:80, respectively [24]. 

The sensitivity obtained in our study for *S. mansoni* IgG-ELISA/NovaLisaTM was 71.2%. Beltrame et al. assessed the accuracy of several serological tests on the basis of microscopy results and obtained a sensitivity of 82% for ELISA [16]. In the study by Kinkell et al., the sensitivity was 64.9% for NovaTec ELISA [22].

The test that has shown the highest concordance with microscopy in the detection of schistosomiasis in our study was the Schistosoma ICT IgG-IgM immunochromatographic test, with a sensitivity of 94.7%. Schistosoma ICT IgG-IgM^®^ is a rapid test that simultaneously detects IgG and IgM antibodies. In schistosomiasis, IgM levels peak at around 12–16 weeks after infection, while IgG peaks at around 20 weeks [18]. Therefore, the capacity of detecting both IgM and IgG could lead to a higher sensitivity of the test by detecting a higher proportion of recent infections and cases of acute schistosomiasis. Further studies in newly infected patients or in early stages of the disease would be necessary to explore how this affects the performance of the test. Several authors have reported results similar to ours. Beltrame et al. in a study in Italy with African migrants, using microscopy as the gold standard, reported a sensitivity of 94% [16]. Leblanc et al. found a sensitivity of 100% for Schistosoma ICT IgG-IgM^®^ in their study [10]. In a recent publication by Hoermann et al., ICT showed a sensitivity of 100% in patients with confirmed schistosomiasis, irrespective of species as *S. mekongi* and *S. japonicum* infections were included in addition to *S. mansoni* and *S. haematobium* [25]. 

There have also been a few studies in schistosomiasis-endemic countries that have evaluated the diagnostic performance of Schistosoma ICT IgG-IgM. Two of them, one in Nigeria for the detection of urinary schistosomiasis using a Western blot (SCHISTO II WB IgG, LDBIO Diagnostics) as the gold standard [30] and one in Zambia for the detection of *S. mansoni* and *S. haematobium* infections using Kato-Katz and urine filtration [31], showed sensitivities of 94.9 % and 100 %, respectively. 

When the results were analysed according to the different *Schistosoma* species, the sensitivity data obtained with IHA and ELISA were higher for *S. mansoni* infections (100% and 82.1%) than for *S. haematobium* (28.6% and 65.7%). However, no differences between species were found when ICT was used (93.3% for *S. mansoni* and 94% for *S. haematobium*). These data are similar to those found in Italy by Beltrame et al. [16], as they obtained sensitivity values of 84% for ELISA and 94% for ICT when considering only *S. mansoni*, and 79% for ELISA and 94% for ICT for *S. haematobium*. In our study, for IHA the data show a much better sensitivity in detecting *S. mansoni* than *S. haematobium*, although the results are limited by the small sample size. In any case, Van Gool et al. [24] suggested that reducing the cut-off titre from 1:160 to 1:80 would strongly increase the sensitivity of IHA (from 88% to 94.7% for *S. mansoni* and from 80% to 92% for *S. haematobium*) with only a slight drop in specificity.

Both *S. mansoni* IgG-ELISA/NovaLisaTM and Schistosoma ICT IgG-IgM showed 100% sensitivity in the diagnosis of *S. intercalatum*/*guineensis* infections, although the number of patients was very small in our series.

Due to the tropism of the different schistosome species, the sensitivity data for UG schistosomiasis are similar to those obtained for *S. haematobium* and those for HI schistosomiasis to those for *S. mansoni*.

Regarding the patients with a false negative serological result, 89.6% were obtained with ELISA, 5.2% with IHA and 5.2% with ICT. Consistent with the literature, the majority of cases, 72.3%, corresponded to *S. haematobium* infections [22]. Marchese et al. reported a proportion of false negatives of 17.5%, of which, 61.1% were *S. haematobium* infections [8]. This is probably due to the fact that most serological tests use antigens against *S. mansoni*. False negative results also occur more frequently in acute or recent infections when the presence of antibodies is not yet detectable (window period), in individuals with late seroconversion (up to 6 months delay) [22] or in those with a low level of antibody response [32]. For such reason, and in order to increase the sensibility of the diagnostic methods, some authors recommend the use of two or more assays in parallel [22]. In addition, in adults in endemic areas where the intensity of infection is generally lower than in young people [6], a decreasing antibody response may occur as a consequence of repeated exposures to schistosome cercariae [21,33]. On the other hand, using a confirmatory test in patients at risk of co-infection by other tissue-invasive helminths would allow to reduce the number of false-positives due to cross-reactivity.

The main limitations of our study derive, first, from its retrospective nature. Second, from the small sample size, especially in the case of IHA testing. Third, it has to be considered a potential technical variability in the procedure of the tests over the years analysed in our study. Fourth, it is possible that in some of the cases diagnosed by biopsy, eggs were already dead because of previous treatments received in home-countries or because of natural death. Nevertheless, in our case, invasive procedures in order to take biopsies (mainly cystoscopies and rectosigmoidoscopies) were merely indicated when active disease was clearly suspected. In the case of urogenital schistosomiasis, biopsies were taken only when hematuria or suggestive bladder nodules in the ultrasound examination were present. For intestinal schistosomiasis, only patients with abdominal pain, diarrhoea or rectal bleeding with no other alternative causes were considered for biopsy. Finally, another weakness is the unfortunate unavailability of archived biological samples to perform the three techniques simultaneously on all samples. On the other hand, the main strengths of our work are the establishment of sensitivity compared to the gold standard of microscopy, the fact that it is a study in real clinical practice, and the high number of patients with confirmed schistosomiasis in our series.

## 5. Conclusions

In conclusion, in view of the recommendations made by international organisations for schistosomiasis serological screening in non-endemic countries, it is necessary to establish protocols for detecting the disease with sensitive tools capable of diagnosing infected individuals in order to provide early treatment and prevent disease progression [16,18,34]. Our study shows that Schistosoma ICT IgG-IgM^®^ immunochromatography has the best performance in real clinical practice, when compared to ELISA and IHA, in both *S. mansoni* and *S. haematobium* infections. Therefore, it could be the test chosen to screen at-risk individuals. Further studies involving a larger number of patients are needed to compare these serological techniques with others that increase the sensitivity of microscopy, such as molecular techniques or antigen detection tests.

## Figures and Tables

**Table 1:** Epidemiological, clinical characteristics and laboratory results of patients with confirmed schistosomiasis.

Total of Patients	N = 405
Mean age in years (range, standard deviation)	27 (11–52) SD 6.46
Gender (number, %)	
Male	379 (93.6%)
Mean time living in Spain in months (range, standard deviation)	35.7 (1–288) SD 37.94
Country of (number, %)	
Mali	199 (49%)
Senegal	92 (22.7%)
Mauritania	33 (8.1%)
Equatorial Guinea	16 (4%)
Guinea Conakry	15 (3.7%)
Gambia	12 (3%)
Guinea Bissau	11 (2.7%)
Ghana	11 (2.7%)
Burkina Faso	7 (1.7%)
Ivory Coast	5 (1.2%)
Nigeria	3 (0.7%)
Sierra Leona	1 (0.2%)
Main reason for referral (number, %)	
Macroscopic hematuria	118 (29.1%)
Abdominal pain	107 (26.4%)
Eosinophilia	68 (16.8%)
Microscopic hematuria	12 (3%)
Anemia	4 (1%)
Laboratory tests results (mean, standard deviation)	
Haemoglobin (gr/dL)	14.7 (1.57)
Total eosinophils (Eo/µL)	640 (689.95)
Platelets (Plt/µL)	224 × 103 (68.57)
IgE (IU/L)	2725 (4102.14)
*Schistosoma* spp. (number, %)	
Urogenital schistosomiasis (301, 74.6%)	
*S. haematobium* (1)	263 (65%)
*Schistosoma* spp. (2)	38 (9.6%)
Hepatointestinal schistosomiasis (114, 28.3%)	
*S. mansoni*	79 (19.5%)
*S. intercalatum*/*guineensis* (3)	10 (2.5%)
*Schistosoma* spp. (4)	26 (6.4%)

SD: Standard deviation.

**Table 2:** Sensitivity of serological tests for the detection of *Schistosoma* infection.

Diagnostic Test	No. Positive/Total Number of Patients Tested (%)	No. Negative/Total Number of Patients Tested (%)
*S. mansoni* IgG-ELISA	215/302 (71.2)	87/302 (28.8)
Bilharziose Fumouze IHA^®^	4/9 (44.4)	5/9 (55.6)
Schistosoma ICT IgG-IgM^®^	89/94 (94.7)	5/94 (5.3)

**Table 3:** Sensitivity of serological tests according to localisation.

Diagnostic Test	No. Positive/Total Number of Patients Tested (%)	
	Urogenital Schistosomiasis (N = 292)	Hepatointestinal Schistosomiasis (N = 104)
*S. mansoni* IgG-ELISA	143/216 (66.2)	70/83 (84.3)
Bilharziose Fumouze IHA^®^	2/7 (28.6)	1/1 (100)
Schistosoma ICT IgG-IgM^®^	65/69 (94.2)	19/20 (95)

**Table 4:** Sensitivity of serological tests according to *Schistosoma* species.

Diagnostic Test	Nº Positive/Total Number of Patients Tested (%)		
	*S. haematobium*	*S. mansoni*	*S. intercalatum*/*guineensis*
*S. mansoni* IgG-ELISA	119/181 (65.7)	46/56 (82.1)	3/3 (100)
Bilharziose Fumouze IHA^®^	2/7 (28.6)	1/1 (100)	-
Schistosoma ICT IgG-IgM^®^	63/67 (94)	14/15 (93.3)	4/4 (100)

## Data Availability

The data that support the findings of this study are available from the corresponding author upon reasonable request.
